# LMdist: Local Manifold distance accurately measures beta diversity in ecological gradients

**DOI:** 10.1093/bioinformatics/btad727

**Published:** 2023-12-07

**Authors:** Susan L Hoops, Dan Knights

**Affiliations:** Department of Computer Science and Engineering, College of Science and Engineering, University of Minnesota, Minneapolis, MN 55455, United States; Department of Computer Science and Engineering, College of Science and Engineering, University of Minnesota, Minneapolis, MN 55455, United States; BioTechnology Institute, College of Biological Sciences, University of Minnesota, Saint Paul, MN 55108, United States

## Abstract

**Motivation:**

Differentiating ecosystems poses a complex, high-dimensional problem constrained by capturing relevant variation across species profiles. Researchers use pairwise distances and subsequent dimensionality reduction to highlight variation in a few dimensions. Despite popularity in analysis of ecological data, these low-dimensional visualizations can contain geometric abnormalities such as “arch” and “horseshoe” effects, potentially obscuring the impact of environmental gradients. These abnormalities appear in ordination but are in fact a product of oversaturated large pairwise distances.

**Results:**

We present Local Manifold distance (LMdist), an unsupervised algorithm which adjusts pairwise beta diversity measures to better represent true ecological distances between samples. Beta diversity measures can have a bounded dynamic range in depicting long environmental gradients with high species turnover. Using a graph structure, LMdist projects pairwise distances onto a manifold and traverses the manifold surface to adjust pairwise distances at the upper end of the beta diversity measure’s dynamic range. This allows for values beyond the range of the original measure. Not all datasets will have oversaturated pairwise distances, nor will capture variation that resembles a manifold, so LMdist adjusts only those pairwise values which may be undervalued in the presence of a sampled gradient. The adjusted distances serve as input for ordination and statistical testing. We demonstrate on real and simulated data that LMdist effectively recovers distances along known gradients and along complex manifolds such as the Swiss roll dataset. LMdist enables more powerful statistical tests for gradient effects and reveals variation orthogonal to the gradient.

**Availability and implementation:**

Available on GitHub at https://github.com/knights-lab/LMdist.

## 1 Introduction

Beta diversity is a standard component of microbial and community ecology analysis. Ordination plots can be found in many microbiome and ecology publications. Geometric anomalies like the arch or horseshoe effects are prevalent in ordination plots of datasets containing an ecological gradient. Ecological gradients could include geochemistry, temperature, altitude, time, or other continuous factors. The arch effect displays samples along the gradient in a curved formation during ordination such that the gradient no longer appears 1D. The horseshoe effect, also referred to as the Guttman effect, is the more extreme relative of the arch effect and displays the samples along an ecological gradient in, as the name suggests, a horseshoe-like shape. Thus, in the horseshoe effect, ends of the gradient appear attracted to one another (Camiz 2005). In the early 1980s, ecologists hypothesized the extreme arch was a mathematical artifact obscuring ecological gradients (Hill and Gauch 1980), adopting the term “horseshoe” from popular political theory discourse.

**Figure 1.**
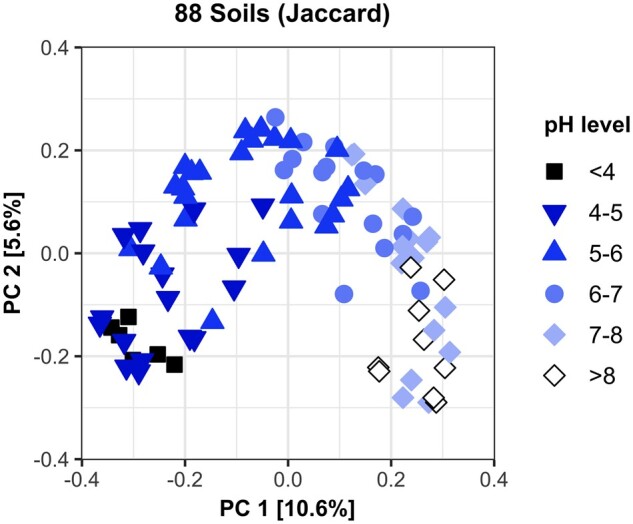
PCoA with Jaccard distances of soil samples taken along a geochemical pH gradient. An arch in the data is clearly visible, making the ends of the pH gradient appear closer than expected.

The arch and horseshoe effects are of concern for beta diversity analyses because they misrepresent the true ecological distances between samples lying along ecological gradients. Figure 1 illustrates an arch in soil samples taken along a pH gradient (Lauber *et al.* 2009). The pH gradient is clearly visible along the arched display of samples, but the arch makes the ends of the gradient (black squares for acidic pH, white diamonds for basic pH) appear almost as closely related to each other as to the middle of the pH gradient. It has been shown previously that this arch phenomenon is not caused by the ordination or visualization (Morton *et al.* 2017), but is instead a problem with the pairwise dissimilarities generating the ordination. Pairwise dissimilarities are the foundation of distance-based statistical analyses for beta diversity, so statistical outcomes may be weakened by pairwise measures misrepresenting sample relationships.

Despite a relatively high frequency of arches and horseshoes present in beta diversity analyses, these artifacts remain relatively underexplored and misunderstood in microbial ecology literature. A limited body of prior work has sought to explain and exemplify the problem as it appears. Ecologists Podani and Miklós explored the tendency of various distance and dissimilarity measures to result in a horseshoe shape in PCoA plots (Podani and Miklós 2002). Their findings indicated that researchers should select distance and dissimilarity measures which do not result in the arch, but their work lacks specificity about the causes or a notable solution. Diaconis *et al.* (2008) indicate some arches may be true representations, but that PCoA is limited in portraying them, and that manifold learning methods for ordination are better suited to data portraying an arch or horseshoe. Still, reasoning about the origin of the arch artifact was lacking until the work of Morton *et al.* (2017) pointed to sparsity of microbiome datasets as a possible source. Morton *et al.* (2017) demonstrate the limitations of distance and dissimilarity metrics in sparse datasets, although their solution, the Earth Mover Band Aware Distance (EMBAD) metric, is designed to rely upon known metadata in adjusting the ordination. EMBAD starts by sorting the samples according to a predetermined environmental variable, then simulating the flow of the taxonomic features between the samples along this ordered list. The EMBAD metric resolves the arched geometry with this adjustment but requires foreknowledge of the ecological gradient and direct use of this gradient in evaluating pairwise distances.

Building on this existing body of work, we hypothesize that distance and dissimilarity metrics have a bounded dynamic range in describing pairwise relationships of a dataset, leading to an oversaturation of large pairwise measures. This effect is caused by samples that are sufficiently separated along the ecological gradient such that they have few or no species in common. Pairwise relationships are more reliable with many species in common, so samples from opposite ends of the gradient will instead have less informative pairwise distances to distinguish them. Upper bounds are known for some popular measures, such as Bray–Curtis and Jaccard dissimilarity which are restricted between zero and one. In these measures, counts of a subset of features will never exceed the total feature counts for a sample. Distance metrics, among them Euclidean distance, Aitchison distances, and UniFrac distances, are unbounded but may oversaturate in the same manner as bounded measures when there are few features in common. Euclidean distances are already known to be a poor choice for sparse datasets, common for microbial ecology (Aggarwal *et al.* 2001). When these large pairwise distances or dissimilarities become oversaturated, ordination algorithms may be limited in differentiating samples in a reduced dimensional space, leading to arches and horseshoes.

Given our hypothesis that the bounded dynamic range of beta diversity measures cause arch and horseshoe effects, we propose Local Manifold distance (LMdist) for adjusting distances and dissimilarities to overcome the limited range of ecological distance measures in sparse datasets. LMdist borrows concepts from graph theory and statistics to better represent the high dimensional geometries of gradient-based datasets. LMdist not only corrects arches and horseshoes in ordination plots but improves the reliability of the underlying pairwise measures for downstream analyses such as statistical hypothesis testing.

## 2 Materials and methods

### 2.1 Local Manifold distance (LMdist) algorithm

**Figure 2.**
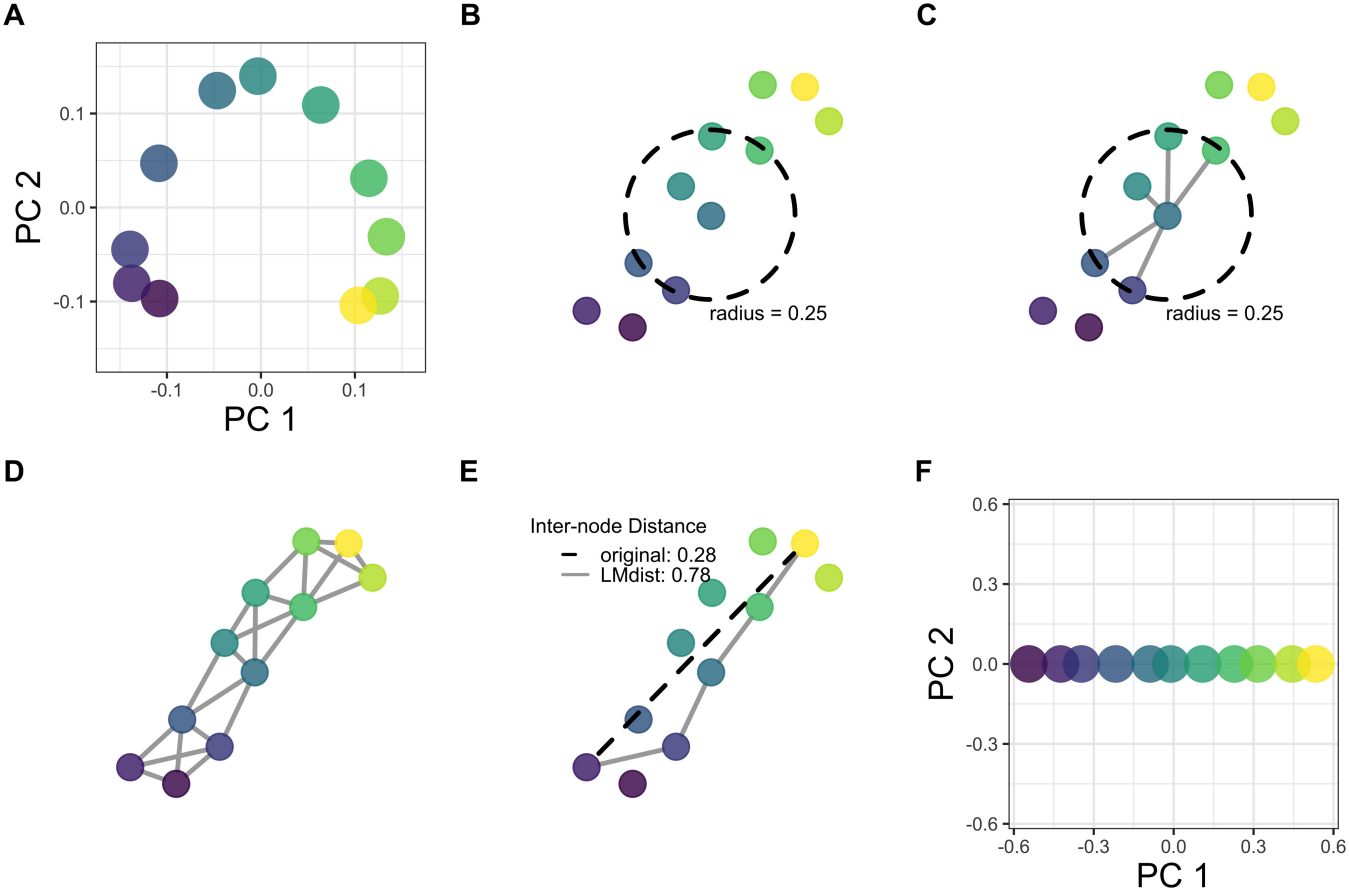
Visual representation of the LMdist algorithm. (A) Simulated samples appearing in an arch during PCoA with Bray–Curtis distances, colored by a simulated ecological gradient. (B) We represent these same samples as isolated nodes and define a neighborhood size by a radius parameter, then (C) add undirected edges between nodes within the parameter-defined neighborhood. (D) Continue to add edges between nodes in common neighborhoods until the graph is fully connected, all nodes can be reached by all other nodes. (E) This graph of nodes and edges is traversed to create adjusted pairwise values, which become the new input for ordination. (F) The resulting ordination can resolve the linear gradient in PCoA.

The LMdist algorithm represents the high dimensional beta diversity matrix as a graph, then measures distances between points along the graph-defined manifold. Figure 2 visually depicts the algorithm on a small simulated gradient dataset. Beginning with an undirected graph of nodes, representing samples, edges are drawn between sample nodes and their parameter-defined neighbors. Each edge is weighted by the distance/dissimilarity between samples at either end, compatible with any pairwise beta diversity measure. Some pairwise edges will be omitted from the graph if they are above the parameterized maximum value, called the neighborhood radius. The graph is then traversed to determine adjusted pairwise values. These adjusted values become the new input for ordination and subsequent statistical analysis, resolving the arch in ordinal plots and enabling more powerful linear regression.

#### 2.1.1 Input pairwise measures form a connected graph

The only required input for the LMdist algorithm is the original pairwise beta diversity matrix; the radius parameter allows for further fine-tuning of the algorithm. The algorithm is compatible with any pairwise measure, and LMdist will adjust these measures to produce a new set of pairwise distances. The neighborhood radius parameter determines which input pairwise values will be included in forming the undirected graph (Fig. 2B and C). For example, given a radius value of 0.25, only pairwise values under 0.25 will be used to connect sample nodes in the graph. Therefore, we can think of the radius parameter as the radius of a sphere in the original beta diversity space, centered at any given sample node. All other sample nodes falling within the bounds of this sphere will be connected to the centroid node as they are considered “neighbors.” Each sample node will be the centroid for this process, until all nodes have been considered and connected to any neighbors to form edges in the graph (Fig. 2D).

After the neighborhood edges have been determined per node using a given radius, the resulting graph may be a disconnected graph. This is expected when samples have not been collected evenly along a gradient, or if the chosen radius is small. If disconnected, a minimum spanning tree (MST) is computed using original pairwise distances for all sample nodes, which traverses all nodes in the shortest possible path. Comparing this MST to the graph of neighbor edges, we can borrow minimal edges from the MST to make the graph fully connected. As a result, we will have a fully connected graph where the minimum possible number of edges exceed the radius parameter, as needed to fully connect the graph.

#### 2.1.2 Recalculate pairwise distances by traversing the graph

This connected graph is used to recalculate pairwise distances/dissimilarities between samples. We calculate a single pairwise distance by traversing from one sample node along any available edges to the other node, in the shortest possible path (Fig. 2E). The adjusted pairwise value is then the sum of all edges traversed in this shortest path between two nodes. This traversal is completed for all pairs of sample nodes, such that we have adjusted pairwise values for any sample relationships exceeding the trusted, radius-defined neighborhood.

#### 2.1.3 Parameters can fine-tune results of LMdist

Fine-tuning of LMdist can be achieved by adjusting the radius parameter, calibrating optimization criteria, or smoothing results via a Gaussian weighting. A large radius, close to the maximum distance, results in a very small adjustment to the original distance matrix. Conversely, a very small radius will cause the largest adjustment, because distant nodes will be connected in the graph only by traversing many intermediate nodes.

By default, the algorithm calculates LMdist using 50 different radii, picking the largest radius which meets two optimization criteria. First, any radius which results in a graph with an average degree, the average number of connections per node, <10% of the number of samples (parameterized as “phi” with default 0.10) will be excluded from the results of LMdist. This limitation is intended to avoid overfitting, resulting in a distorted graph that might exaggerate the distances between samples. The second criterion is a multi-objective function which allows LMdist to guess the best radial value for a given dataset. This multi-objective optimization maximizes both the radius and a correlation between the LMdist-adjusted distances and the Euclidean distances in resulting ordination (PCoA) space, using the first *n* dimensions, up to 10, accounting for >80% variance. To give priority to trusting more original distances, a correlation for a smaller radius must exceed the previous best correlation by a parameterized value of epsilon (default 0.05).

The multi-objective function works for many cases, but still places confidence in a single radius value. We therefore implemented an optional parameter (“smooth” defaulted to False) for smoothing results of multiple radii. If smoothing is preferred, a Gaussian weight centered at the chosen radius is applied to the results of up to 50 radii, such that many radial values will influence the results, generating a smoothed version of the corrected pairwise distances.

#### 2.1.4 LMdist produces an adjusted distance matrix

The result of the LMdist algorithm, implemented in R (R Core Team 2021), is an adjusted pairwise distance object, the same format as the output of the vegdist function in the vegan package (Oksanen *et al.* 2022). These adjusted values can replace the original pairwise distance matrix in subsequent beta diversity analysis. In ordination, the adjusted values become the new input for ordinal methods accepting pairwise measures, such as PCoA or NMDS. Results in a simulated dataset and case studies have shown that LMdist reduces the arched appearance of the data, and if an environmental gradient is present LMdist better resolves the gradient, revealing variation orthogonal to the gradient in other ordinal dimensions.

#### 2.1.5 Use cases have oversaturated distances

LMdist can be used with any pairwise distance matrix but is best utilized for uncovering gradients. While some gradients may already be well represented, an arch or horseshoe in ordination tends to appear when the pairwise distance matrices become oversaturated. We define oversaturated distances as a large left skew in the distribution of pairwise distances, because large distances are relatively oversaturated compared to the smaller pairwise distances. This oversaturation indicates a disagreement between the sampled gradient and the pairwise sample distances, as is demonstrated in implementation with a simulated dataset to follow (Fig. 4). Therefore, we propose LMdist is most appropriately used in studies with a long underlying gradient or oversaturated distances, as these samples will be better represented when accounting for the underlying manifold. Case-control studies may not need adjustment by LMdist, most importantly if the pairwise distances are not oversaturated with large values.

### 2.2 Simulated dataset

For initial validation of this algorithm, we created a simulated dataset of taxonomic features evenly distributed along an artificial gradient, mimicking a gradient-driven ecosystem. The final simulated dataset contains 50 samples and 231 taxonomic features. Noisy samples added to the dataset illustrate the sensitivity of the algorithm, which has remained stable in uncovering the gradient.

#### 2.2.1 Coenoclines emulate species abundance along a gradient

**Figure 3.**
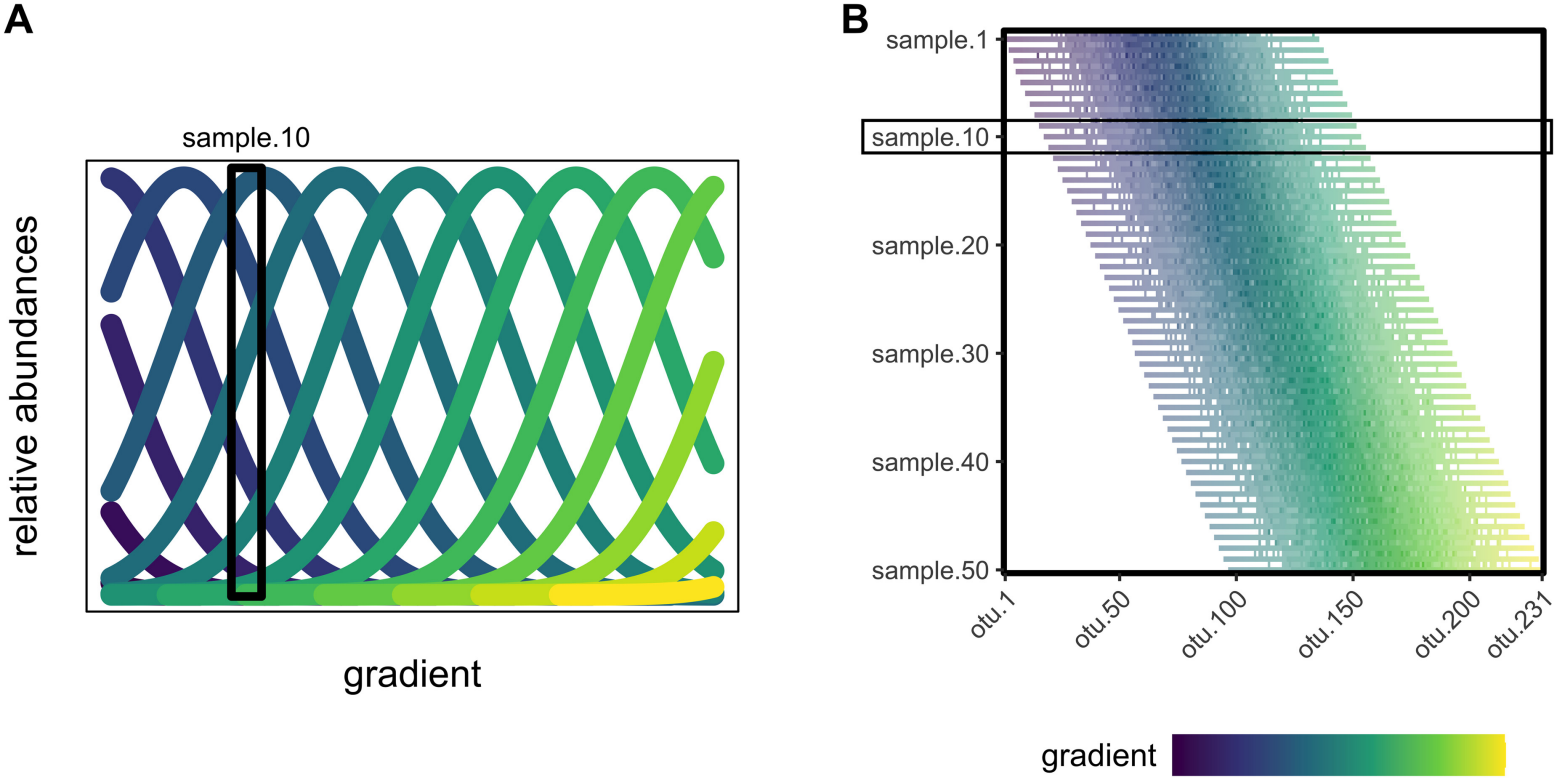
Creation of simulated dataset. (A) Coenoclines representing roughly every 10th taxonomic feature distributed along the simulated gradient, annotated to show how sample 10 would be collected. Sample 10 would be comprised of the amounts of each feature as seen in the visual cross section of the feature coenoclines. (B) Heatmap of samples collected, displayed in order with the noise samples preceding each original sample.

In creating a simulated dataset we utilized coenoclines, representations of species response functions along some gradient (Palmer 2013). We presumed a unimodal response curve for each species along the gradient, supported by further exploration of the soil dataset (Supplementary Fig. S1). For our simulated gradient, we distributed artificial species features evenly along a gradient, using Gaussian coenoclines (Fig. 3A). For an example gradient length of 1000 (arbitrary units), the artificial species distributions are simulated as Gaussian curves centered 10 steps apart with a standard deviation of 150, allowing for density of overlap between species. Samples are obtained from these coenoclines by sampling values from the densities of the Gaussian curves at a given point, attributing the amounts of every artificial species at that point in the gradient to the sample (Fig. 3A). A total of 50 samples are taken, evenly distributed along the full gradient length. The absolute heights of the Gaussian coenoclines are arbitrary because samples are normalized to relative abundances before analysis.

#### 2.2.2 Sampling noise from a Dirichlet distribution

To add noise to the simulated dataset, each mixture of sampled species values is used as the prior for a Dirichlet distribution, the generative distribution of the multinomial distribution and the multivariate generalization of the Beta distribution (Ng *et al.* 2011), producing 50 additional samples as noise, mimicking the original samples (Fig. 3B). We chose the Dirichlet distribution because it allows for fine-tuning the degree of noise and because microbial ecology samples have been modeled previously using a Dirichlet distribution (Harrison *et al.* 2020).

## 3 Results

### 3.1 Simulated gradient

**Figure 4.**
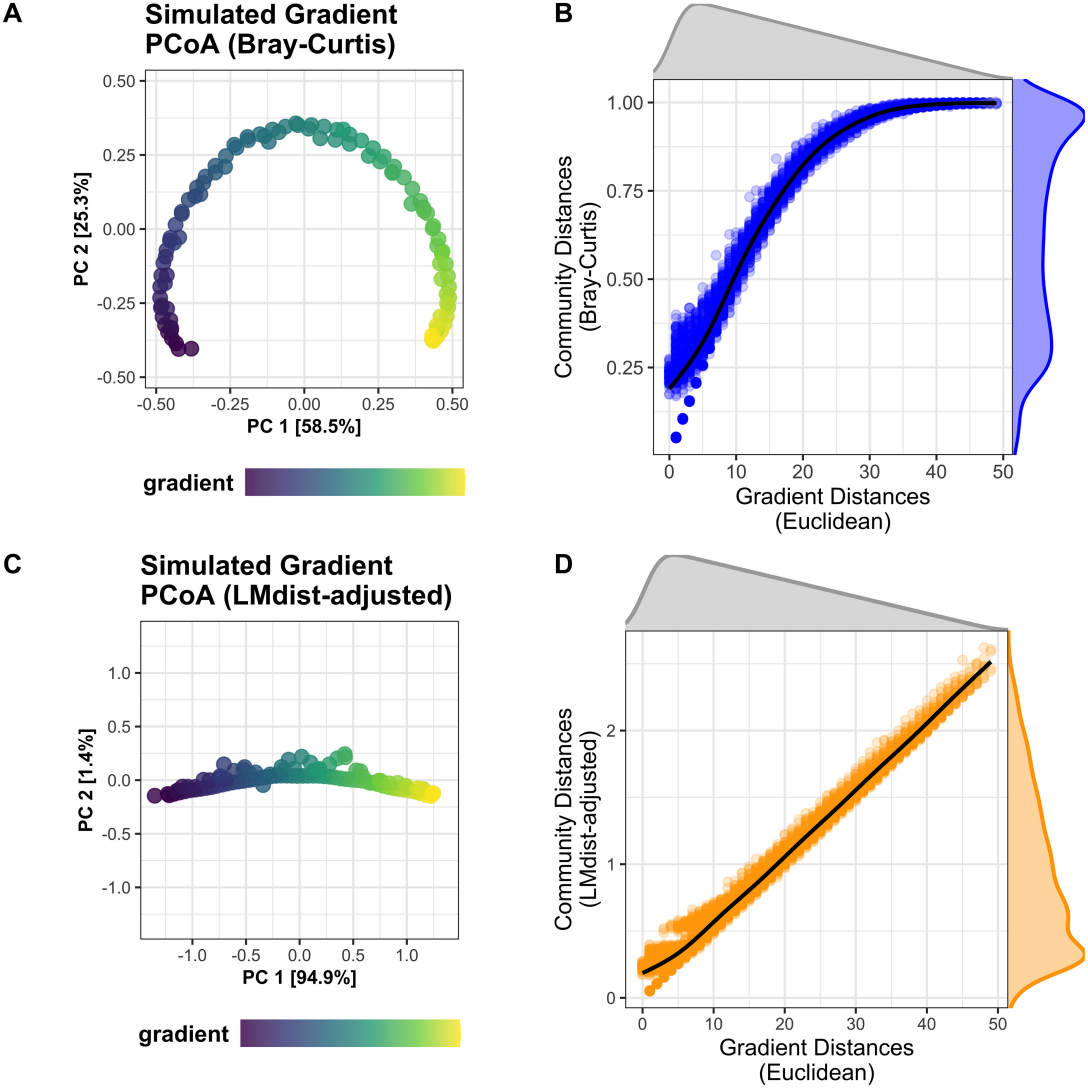
LMdist applied to the simulated gradient. (A) PCoA with Bray–Curtis distances of 100 simulated samples, colored by approximate position on gradient. (B) Comparison plot of community distances (Bray–Curtis) and gradient distances (Euclidean). We can see the community distances plateau at the top of the gradient, causing a mismatch between the density of community and gradient distances. (C) PCoA with LMdist-adjusted distances, where the horseshoe is largely resolved along the first principal component. (D) The same comparison plot between the adjusted community distances and gradient distances, the distribution of which is better balanced to one another after LMdist is applied.

Figure 4 shows the simulated dataset after application of the LMdist algorithm to adjust distances. In the original Bray–Curtis distances, we can see from Fig. 4B that the Bray–Curtis distances on the *y*-axis are distributed with the opposite skew of the known gradient distances on the *x*-axis, resulting in a plateau rather than the expected *y* = *x* line. This oversaturation of large distances results in the samples forming a horseshoe in PCoA ordination (Fig. 4A). After applying LMdist with a radius of 0.4, the gradient distances and adjusted distances now resemble one another (Fig. 4D) and the gradient is resolved in PCoA along the first principal component (Pearson correlation 0.999, improved from 0.967 prior to LMdist) such that the second axis may now be accounting for noise (Levene’s test of variance, *P *<* *.001) (Fig. 4C).

### 3.2 Simulated Swiss roll

**Figure 5.**
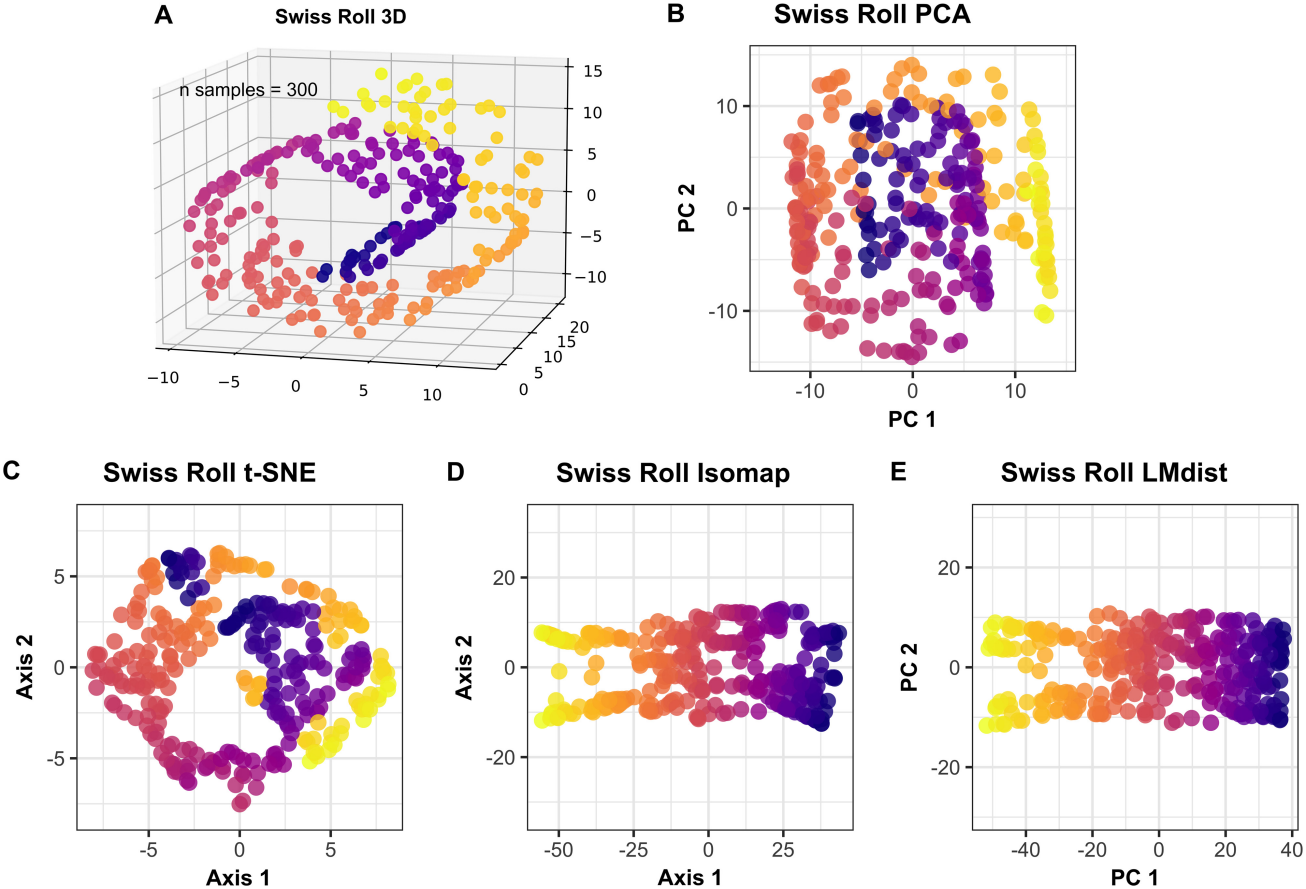
LMdist applied to the Swiss roll dataset. (A) The Swiss roll dataset with 300 samples in the original 3 dimensions, created with the scikit-learn package in Python. (B) PCA of the Swiss roll dataset. (C) t-SNE of the Swiss roll dataset using perplexity 60. (D) Isomap of Swiss roll dataset with neighborhood size 5. (E) LMdist of Swiss roll dataset with neighborhood radius 0.62.

The Swiss roll dataset has been used to exemplify an obvious low-dimensional manifold structure in demonstrating manifold learning approaches. Figure 5 shows 300 samples in a Swiss roll, created using the scikit-learn python package (Pedregosa *et al.* 2011), in the original three dimensions. We then compare to dimensionality reduction by principal component analysis (PCA), t-distributed Stochastic Neighbor Embedding (t-SNE) (Van der Maaten and Hinton 2008), Isomap (Balasubramanian and Schwartz 2002), and LMdist. Fine-tuning of parameters was necessary for all algorithms to obtain the best possible flattening of the manifold in 2D space.

As expected, the manifold learning approaches and LMdist outperform PCA, due to their parameter-defined perplexity and ability to recover some of the underlying structure of the data. LMdist proves particularly useful in two respects compared to these manifold learning approaches. First, LMdist appears to most reliably uncover sample relationships, as it is not overly sensitive to hyperparameters and a series of transformations as is the case of t-SNE, nor does LMdist assume neighborhoods of equal sample sizes as in Isomap. The neighborhood radius used by LMdist therefore resolves the manifold, but also more clearly resolves the variation along the manifold in the second dimension. Secondly, LMdist is advantageous as the only approach to outputs an actual distance matrix, which can then be used for distance-based statistical testing. Typically, the adjusted distance matrix is only implicit within manifold learning algorithms, precluding their use in downstream analysis such as statistical testing of beta diversity.

### 3.3 Case studies demonstrating the arch effect

**Figure 6.**
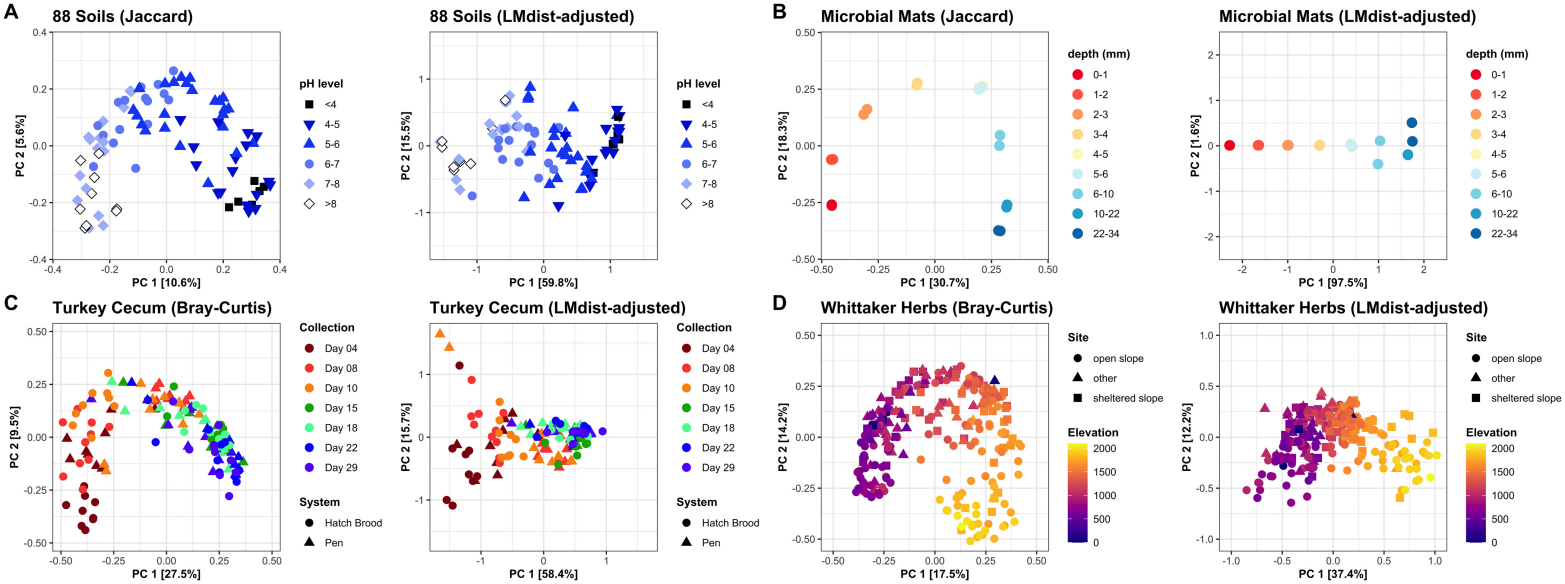
PCoAs before and after adjusting pairwise values with LMdist on default settings, displayed with fixed square perspective. (A) Soil samples along a pH gradient, Jaccard dissimilarity and LMdist with radius 0.952. (B) Microbial mat samples at various depths, Jaccard dissimilarity and LMdist with radius 0.758. (C) Longitudinal Turkey cecum samples, Bray–Curtis dissimilarity and LMdist with radius 0.599 (note: changed epsilon to 0.01 since the default epsilon was too small). (D) Herbs and shrubs community data collected by Robert Whittaker in the Siskiyou Mountains, Bray–Curtis dissimilarity and LMdist with radius 0.93.

To exemplify the usefulness of the LMdist algorithm, we have gathered datasets from a variety of microbiome and ecological studies (Fig. 6). As previously described, oversaturation of the distance and dissimilarity measures is often driven by underlying ecological gradients in the dataset. Therefore, datasets were selected which have a measurable environmental gradient and which exhibited an arch or horseshoe in ordination.

First, we return to the soil samples from across North and South America comprising a pH gradient (Fig. 6A). Lauber *et al.* found these bacterial communities to be significantly correlated with soil pH (ANOSIM, *P *=* *.001; Mantel test Spearman, *r* = 0.73), largely driven by the presence and absence of the phylum *Acidobacteria*, known to favor acidic environments, as well as the phyla *Bacteroidetes* and *Actinobacteria* which prefer comparatively basic environments (Lauber *et al.* 2009). After applying LMdist, we can see that the arch is largely resolved in the visualization, with no change in significance of distance-based testing (ANOSIM, *P* = .001; Mantel test Spearman, *r* = 0.74). Interestingly, though, we find that PC2 now resolves some variation in the data orthogonal to the pH gradient in PC1, correlating with amount of silt clay (Pearson cor = 0.51, *P *<* *.001; PERMANOVA, *P *<* *.001) and annual season temperature (Pearson cor = 0.49, *P *<* *.001; PERMANOVA, *P *<* *.001). While silt clay and annual season temperature were significant prior to LMdist using PERMANOVA (*P *<* *.05), *F*-scores for silt clay, annual season temperature, and pH all increased, implying the effect size is better represented after application of LMdist. This new finding with silt clay and annual season temperature orthogonal to the pH gradient was not mentioned in the original publication and might have been obscured by the arch in the original analysis.

Sampling increasing depths of the hypersaline microbial mats of Guerrero Negro in Baja California Sur provides another gradient analysis on a smaller dataset. The authors confirm that the depths of microbial mats are phylogenetically stratified, as light and oxygen produced by photosynthesis rapidly diminishes with depth (Harris *et al.* 2013). Each depth from the top of the microbial mat to a depth of 34 mm is sampled twice and sequenced with 454 pyrosequencing technology. We can clearly make out that the depths follow a horseshoe pattern in the original ordination (Fig. 6B). Using LMdist, we can resolve the horseshoe while trusting many distances (neighborhood radius 0.758 for Jaccard dissimilarity is chosen by the default algorithm) (Fig. 6B). The correlation of the gradient with the first principal component (Spearman rho = 0.941, *P *<* *.001) improved after application of LMdist without changing significance (Pearson cor = 0.987, *P *<* *.001).

Our next case study is a longitudinal, observational experiment comparing the Turkey gut when raised in an isolated hatch brood system as opposed to a commercial brood pen (Fig. 6C) (Miller *et al.* 2021). Ignoring the first day samples since they formed a separate cluster, we can resolve the longitudinal changes using LMdist. The authors found that beta diversity significantly separated the samples by the collection date (PERMANOVA, F-score = 23.8, *P *<* *.001) and upbringing groups (Mantel test, *r* = 0.38, *P *=* *.001). After applying LMdist, we found the collection day gradient improved the effect in PERMANOVA found between collection day and pairwise measures (F-score = 57.63, *P *<* *.001) without changing the significance found for raising group (PERMANOVA, *P *=* *.007).

Finally, we evaluate the usefulness of LMdist applied to herb and shrub community data collected by Robert Whittaker in the Siskiyou Mountains along an elevation gradient (Fig. 6D) (Whittaker *et al.* 2022). Restricting to just the communities in diorite soil, the elevation gradient forms an arch in PCoA of Bray–Curtis dissimilarities. Again, the effect size of the elevation gradient (PERMANOVA, F-score = 36.8, *P *=* *.001) is improved after applying LMdist (PERMANOVA, F-score = 92.4, *P *=* *.001).

These case studies exhibiting gradient-driven arches and horseshoes demonstrate how LMdist may better resolve gradient effects. Resolving gradients along one principal component enables more powerful linear statistical tests of gradient impact. LMdist also uncovers other variation orthogonal to the gradient, presenting some novel findings previously obscured by horseshoes. The optional gaussian smoothing parameter in LMdist minimally alters output for these case studies (Supplementary Fig. S2), implying LMdist with both a single radius and smoothing present similar, reasonable solutions.

In cases where an underlying gradient is not present, such as a clustered dataset, we have found that in both simulated and real clustered data LMdist does not resolve an arch unless the original pairwise beta diversity values are truly oversaturated (Supplementary Fig. S3) (Whittaker 1956, Yatsunenko *et al.* 2012, Vangay *et al.* 2018). One can test for oversaturation by evaluating if the data presents a left skew in the distribution of pairwise distances. If no left skew is present, we expect there is no long underlying manifold, nor oversaturation of pairwise values. When we apply LMdist to simulated (Supplementary Fig. S3C) practical (Supplementary Fig. S3D and E) examples without oversaturation, we find LMdist chooses not to adjust pairwise distances as they are already the best representation of sample relationships. These experiments indicate LMdist is robust to overfitting and will not adjust already reliable pairwise values.

## 4 Discussion

We have demonstrated the usefulness of LMdist in a simulated gradient and in case studies of real environmental gradients across a range of published datasets. The simulated gradient can be nearly perfectly resolved with LMdist-adjusted distances. In various case studies, we find that LMdist can not only visually resolve the arched geometry of the data but can improve statistical outcomes in distance-based tests. LMdist is best used when pairwise beta diversity measures are oversaturated, presenting a left skew in the distribution of distances. These cases are typical of sampling a gradient or finding an arch in ordination, not as common for case-control or cohort studies. However, LMdist is also designed to resist overfitting, only correcting oversaturated pairwise values of which there may be none in a clustered dataset (Supplementary Fig. S3). In several gradient-based case studies, LMdist-adjusted distances improved the correlation of a known continuous gradient with the first axis of ordination, enabling more reliable use of linear regression approaches.

By adjusting distance/dissimilarity measures with LMdist to decrease saturation of large pairwise distance values, and to increase trustworthiness of distances, ordination plots more accurately portray global relationships in the data. We have also demonstrated that LMdist reveals new information driving variation in the data orthogonal to the gradient, such as the second principal component in the soil dataset (Fig. 6A). Prior to LMdist, the arch had occupied two or more dimensions instead of one, therefore leaving this orthogonal variation obscured. LMdist is therefore a powerful analysis technique in determining other interactions beyond the main environmental drivers in a study, allowing researchers to dive deeper into new explanatory variables. An important feature of this approach is that LMdist is completely unsupervised, not relying on foreknowledge of the gradient driving variation.

LMdist is an efficient, data-driven algorithm for resolving arches and horseshoes driven by underlying gradients. With proper utilization, LMdist has shown promise in enabling more powerful statistical tests between environmental gradients and high-dimensional microbiome datasets. Analysis with LMdist-corrected distances may uncover new ecological variation that is orthogonal to environmental gradients, expanding the findings of previously published works.

## Supplementary Material

btad727_Supplementary_Data

## Data Availability

No new data were generated or analysed in support of this research. All data can be found in previously published works. Software and simulated dataset generation is available at https://github.com/knights-lab/LMdist.
