# Supplementary material for: LMdist: Local Manifold distance accurately measures beta diversity in ecological gradients
Source: Bioinformatics. 2023 Dec 7;39(12):btad727. doi: 10.1093/bioinformatics/btad727 (PMC10713119; doi:10.1093/bioinformatics/btad727)
Supplement: btad727_Supplementary_Data [file btad727_supplementary_data.docx]

**Supplemental Material**

Title: LMdist: Local manifold distance accurately measures beta diversity in ecological gradients.

Authors: Susan L. Hoops, Dan Knights


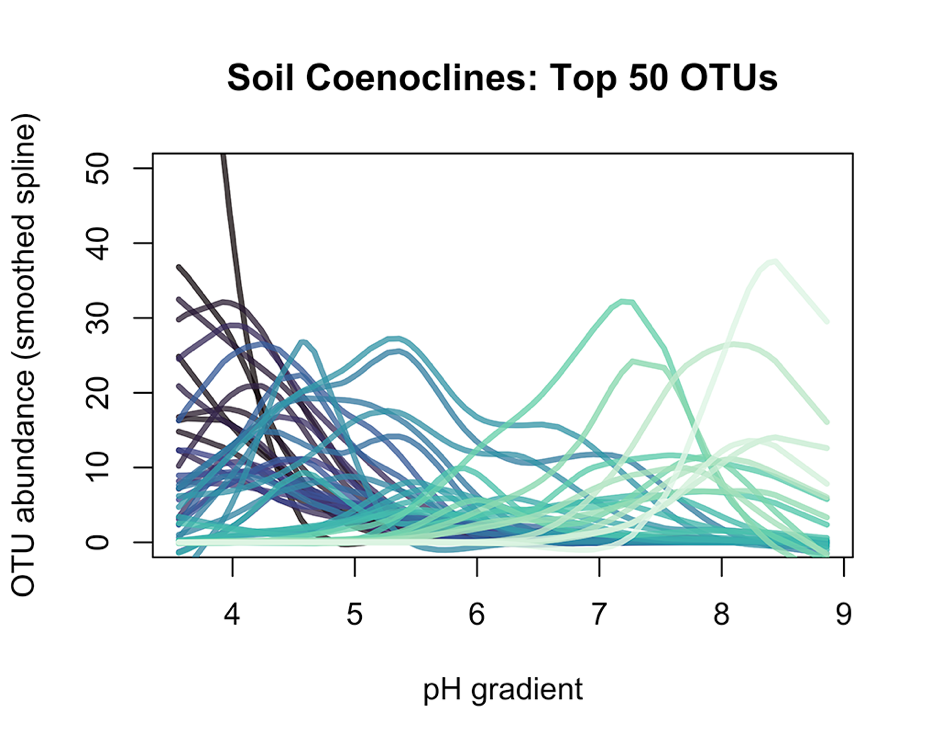


**Figure S1.** Coenoclines from the 88 soils dataset. Only the first 50 most abundant species are displayed, colored by their peak position along the pH gradient from acidic (black, dark teal) to basic (light teal).


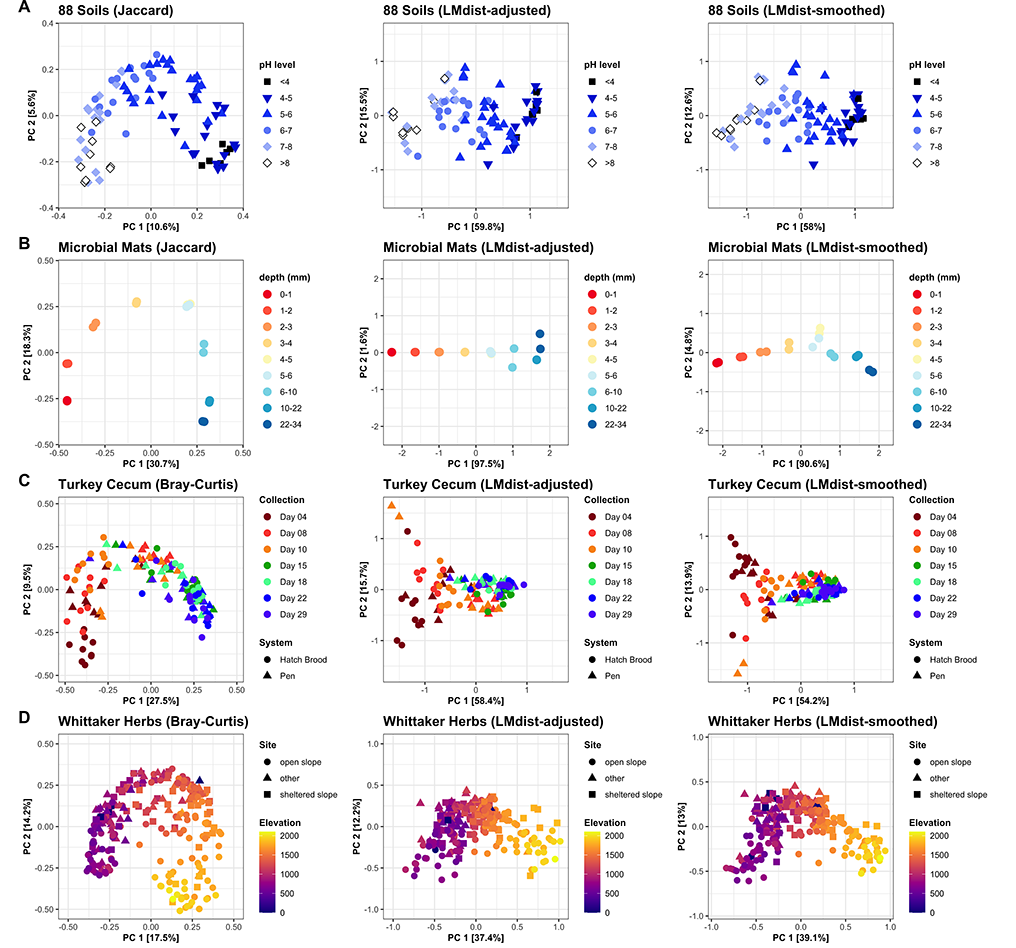


**Figure S2.** Demonstration of the optional smoothing parameter in LMdist using the same case studies as seen in Figure 6: (A) soil samples along pH gradient, (B) microbial mat samples at varying depths, (C) longitudinal turkey cecum samples, and (D) herbs and shrubs from the Siskiyou mountains. The third column uses the smoothing parameter to apply a Gaussian weighted mean to multiple radius values, such that no single radius value is trusted completely in the output pairwise values of LMdist.


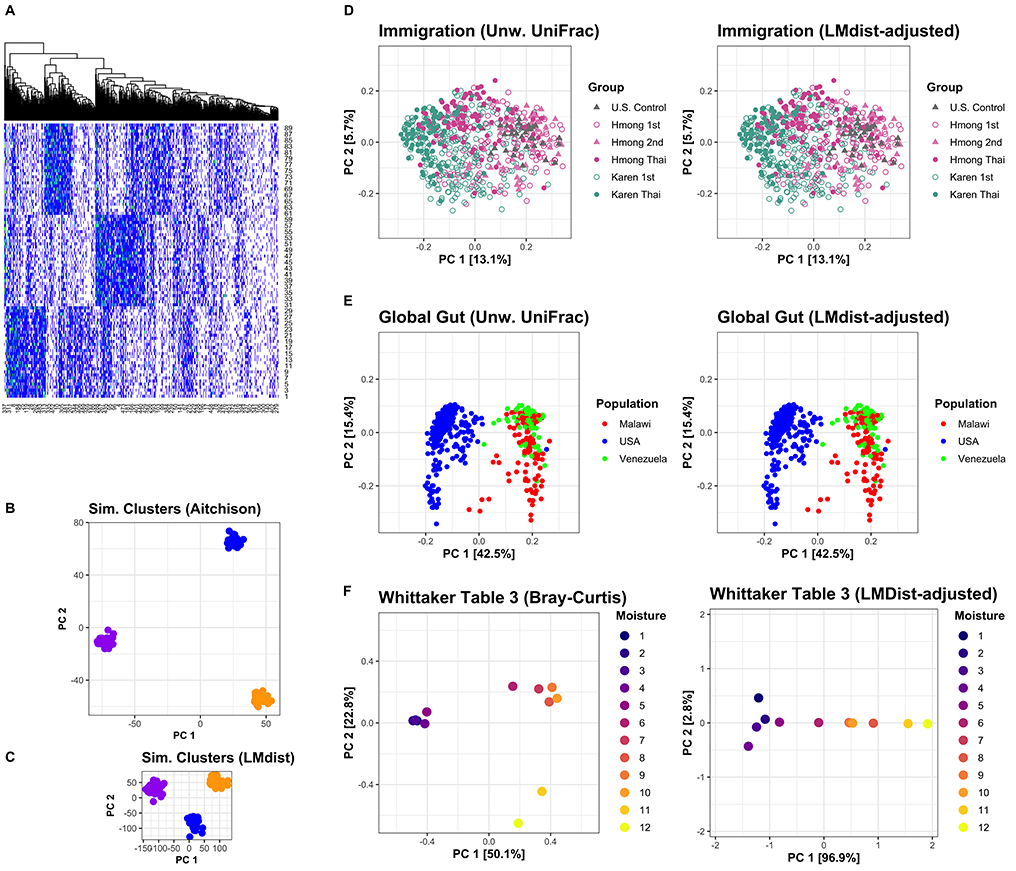


**Figure S3.** Sensitivity of LMdist demonstrated by application to simulated and real data without a dominant gradient effect. (A) The simulated clustered dataset, visualized as a matrix of samples and simulated OTU abundances. Each cluster of 30 samples is comprised of sub-sampling a Dirichlet distribution from a single sample of a beta distribution (alpha = 1, beta = 20). (B) PCoA with Aitchison distance of the simulated clusters. (C) PCoA of after running LMdist on the simulated clusters, but LMdist chose not to adjust the distances using the default parameters, as expected for clusters of samples. (D) Human gut dataset describing U.S. immigration from Thailand (Vangay *et al.* 2018), LMdist (default parameters) does not alter the pairwise distances. (E) Human gut dataset from different continents (Yatsunenko *et al.* 2012), LMdist (default parameters) does not alter the pairwise distances. (F) A small sample from Robert Whittaker’s exploration of the vegetation in the Great Smokey Mountains, where we can LMdist (default parameters, chooses radius 0.633) resolves the original clusters into more of a gradient, seemingly following the environmental gradient of moisture (Whitaker 1956). This study (F) exemplifies clusters which may have an underlying gradient, while the human gut studies (D-E) evidently did not have a gradient to resolve.
